# Increased Cognitive Demands Affect Agility Performance in Female Athletes - Implications for Testing and Training of Agility in Team Ball Sports

**DOI:** 10.1177/00315125221108698

**Published:** 2022-06-15

**Authors:** Daniel Büchel, Alli Gokeler, Pieter Heuvelmans, Jochen Baumeister

**Affiliations:** 1 Exercise Science & Neuroscience Unit, Department Sports & Health, 26578 Paderborn University, Paderborn, Germany

**Keywords:** complex speed, cognition, learning and memory, speedcourt, agility, team sports

## Abstract

Agility, a key component of team ball sports, describes an athlete´s ability to move fast in response to changing environments. While agility requires basic cognitive functions like processing speed, it also requires more complex cognitive processes like working memory and inhibition. Yet, most agility tests restrict an assessment of cognitive processes to simple reactive times that lack ecological validity. Our aim in this study was to assess agility performance by means of total time on two agility tests with matched motor demands but with both low and high cognitive demands. We tested 22 female team athletes on SpeedCourt, using a simple agility test (SAT) that measured only processing speed and a complex agility test (CAT) that required working memory and inhibition. We found excellent to good reliability for both our SAT (ICC = .79) and CAT (ICC =.70). Lower agility performance on the CAT was associated with increased agility total time and split times (*p* < .05). These results demonstrated that agility performance depends on the complexity of cognitive demands. There may be interference-effects between motor and cognitive performances, reducing speed when environmental information becomes more complex. Future studies should consider agility training models that implement complex cognitive stimuli to challenge athletes according to competitive demands. This will also allow scientists and practitioners to tailor tests to talent identification, performance development and injury rehabilitation.

## Introduction

Team ball sport athletes must quickly adapt to ever-changing environmental cues, including changes in one’s direction and speed in response to ball movement trajectories, and opponents’ and teammates’ actions (Sheppard et al., 2006). Young et al. (2015) defined this adaptive skill as “reactive agility,” a subcomponent skill of complex coordination that incorporates and regulates motor, sensory and cognitive behavior during (goal-directed) movements (Baumeister, 2013). Accordingly, Young et al. (2015) suggested that agility combines two sets of abilities: motor abilities involved in speed and change of direction (COD) and cognitive abilities associated with perception and decision-making (Young et al., 2015). Hence, COD reflects pre-planned movements that lack perception-action coupling and involve movements out of the game context (Young, 2021). Conversely, agility also requires cognitively mediated perceptual and consecutive processing and responses to environmental stimuli (Sheppard et al., 2006). Considering the differences between isolated COD movements and agility, it is no surprise that past investigators have reported non-significant correlations between COD movements and agility (Matlák et al., 2016; Scanlan et al., 2014).

Considering the cognitive challenges that a team sports athlete experiences during competition (Huijgen et al., 2015), a shortcoming of current agility testing is that it often relies on simple reaction time paradigms (Pojskic et al., 2018; Sekulic et al., 2019; Spasic et al., 2015) that are only representative of lower cognitive function, such as processing speed (Morral-Yepes et al., 2020). In team sports, more complex mental processes or so-called higher order cognitive or executive functions (Diamond, 2013) are required. These executive functions (EF) refer to the mental abilities needed to coordinate cognitive, emotional, and motor responses as a set of adaptive behaviors. EF allow athletes to successfully and proactively navigate in the environment by shifting thought processes and adapting to changing situational game cues (Jurado & Rosselli, 2007). EF can be categorized into sub-components such as working memory, inhibitory control, and cognitive flexibility (Diamond, 2013). Working memory allows individuals to hold information in mind and work with it mentally, even without cues of its importance. Inhibitory control involves the ability to control attention, behavior, thoughts and/or emotions to cancel strong internal predispositions or external temptations to behave with automaticity. Cognitive flexibility builds on working memory and inhibitory control in that it describes the ability to quickly change a perspective and shift a mental set by inhibiting or deactivating an earlier mindset to entertain and load into working memory a newer view of a problem.

While cognitive abilities contributing to agility performance are critical to sports success in complex situations, their assessment and analysis are underrepresented in past research. To our knowledge, no investigators have yet examined how different levels of cognitive demands may interact with the motor abilities in agility performance. For a comprehensive understanding of both the physical performance and cognitive abilities that contribute to agility performance and how to tailor agility training to individual athletes, further research is needed (Morral-Yepes et al., 2020). Therefore, our two aims in this study were (i) to assess, among a group of female athletes, how agility performance changes in the context of low and high cognitive stimuli, and (ii) to analyze the reliability of low and high cognitive demand tests for agility among these athletes. Hence, we compared two agility tests: (i) one with low demands for simple cognitive functioning (i.e., information processing speed); and (ii) one with high demands for more complex cognitive functioning (i.e., inhibitory control and working memory). To account for systematic test-retest changes in agility performance due to practice effects, we assessed both tests twice within one week and used the second set of test scores for data analysis. We hypothesized that agility performance, expressed by movement speed, would decrease when higher order cognitive functions were required, illustrating the impact that the cognitive component of agility can have on agility performance.

## Method

### Participants

We calculated a required sample size to achieve .9 statistical power at an alpha level of .05 using GPower, Version 3.1 (Faul et al., 2007), and we based assumed effect sizes on Henry et al. (2012) who reported effect sizes of .88 when comparing feint and non-feint stimuli on 24 football players’ reactive agility in a repeated measures, within factors ANOVA design. This calculation led to a required participant sample size of 16. Accounting for possible attrition, we then recruited 22 female team sport athletes for this study (*M* age = 21.9, *SD* = 3.5 years). All participants had been playing a team sport at a regional level (soccer = 12, handball = 3, tennis = 2, field hockey = 2, basketball = 1, volleyball = 1, lacrosse = 1) for an average of 10.5 years at a pace of at least twice per week, and all were recruited at our university. Based on reported sex differences in agility performance (Dos’santos et al., 2018; Sekulic et al., 2013), we recruited only females to increase performance homogeneity in the sample. Before beginning the experiment, we informed all participants about the study and procured their signed informed consent. The study was conducted in accordance with the declaration of Helsinki and was approved by the ethical committee of the affiliated university.

### Testing

**Figure 1.**
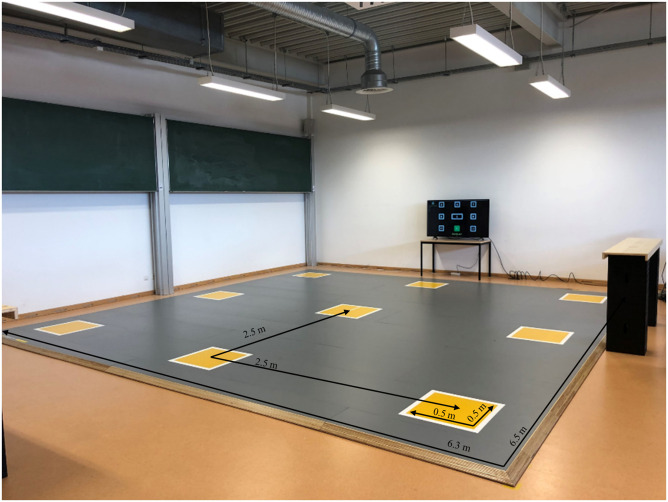
Overview of the Experimental Protocol. *Note*. Participants came in on two days with a break of >48 hours in-between sessions. The same protocols were performed on both days. Each participant performed two trials of change-of-direction speed (COD) test, four trials of simplex agility test (SAT) and four trials of complex agility test (CAT). SAT and CAT were performed in a randomized order.

**Figure 2.**
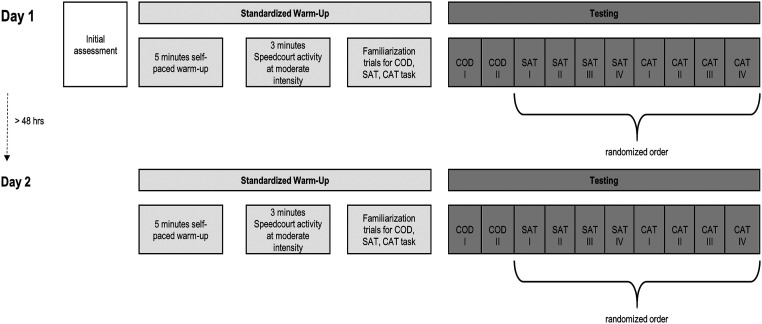
Overview of the Speed Court System Consisting of a Court with Contact Fields (1) and a TV Screen for Stimulus Presentation (2).

All testing in the present study took place within the SpeedCourt system (Globalspeed GmbH, Germany). The SpeedCourt includes a computer linked to a TV screen and pressure sensors placed in a 3-by-3 grid (see Figure 1). The pressure sensor squares are each 40-by-40 cm and equally distributed on a 6.3 m × 6.5 m court. On a display, the participants can see a digital representation of the court. Depending on the test, single squares on the screen light up to show the participant where to run next. As soon as the corresponding square is touched, another square on the screen lights up. Participants performed three test conditions on the SpeedCourt in a randomized order (see Figure 2): (a) two trials of a COD test, (b) four trials of a simple agility test (SAT); and (c) four trials of a complex agility test (CAT).

The COD was used to familiarize participants with the task and to determine a reference value for movement speed on the agility tests. The COD used in this study was described in detail in Düking et al. (2016). Participants had to run as fast as possible on a predefined route of approximately 26.6 m, including seven preplanned turns of 45°–180°. For the SAT, participants started at the center contact field and had to run as fast as possible to a representative contact field shown as a yellow square on the screen. After touching this contact field, participants always had to run back to the center contact field before the next random stimuli was presented. To assure that the task resembled comparable COD patterns for all participants, only the left and right upper and lower corner contact fields lit up. Since the corner contact fields lit up in a randomized order, the agility task was unpredictable. We operationalized the SAT as a low cognitive functioning test of information processing speed.

For the CAT, participants had to run the same pattern as for the SAT. The main difference was a change in the cognitive demands of the task. For the CAT, the square on the screen not only lit up yellow, but there was a 75% likelihood that the screen would also present an accompanying blue, pink or green color frame around the square. Fields solely lit as yellow required the participants to run to the corresponding field, but squares with additional blue, pink and green frames around the field required participants to perform an additional task as follows:

• blue = “run to the indicated contact field”;
• pink = “run to the front-right contact field”;
• green = “run to the front-left contact field”.

**Figure 3.**
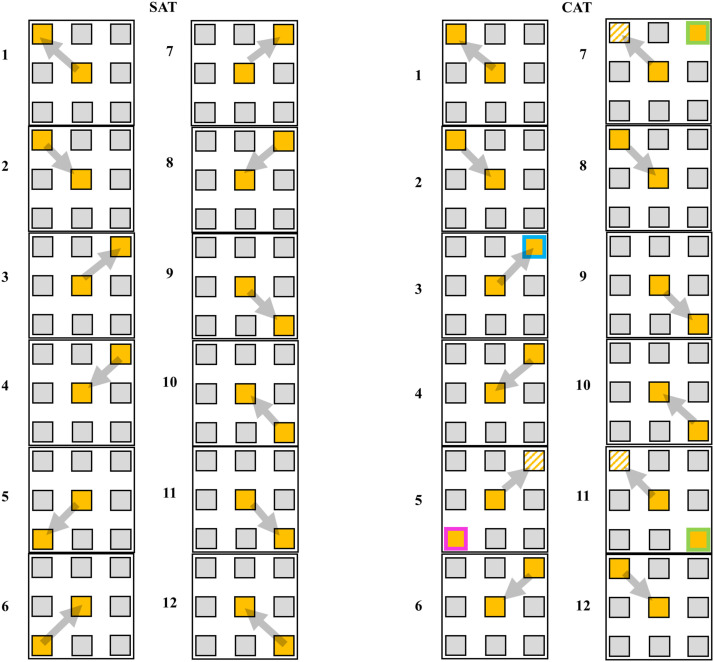
Exemplary Overview of Stimulus Sequences of the Simple Agility Test (SAT) and the Complex Agility Tests (CAT). *Note*. Both tests contained 12 changes of direction, from which the even ones appeared randomized in the corner squares. The SAT only contained easy reaction stimuli (yellow fields), the CAT also included three additional colors which imposed higher neurocognitive demands (blue frame=“run to indicated field”; pink frame = “always run to right top field”; green frame = “always run to left top field”).

Thus, the CAT required more complex cognitive functioning that involved working memory and inhibitory control. For example, participants had to store information in working memory and, in the case of pink and green frames, they had to inhibit their associations with the yellow visual stimulus (= square) and only respond to the demands of the colored frame. An example of stimulus sequences for the CAT and SAT are provided in Figure 3.

For both SAT and CAT, the total distance to cover was approximately 42.4 m and included 12 changes of direction. Among these 12 changes of direction, the six towards the outside fields were unpredictable as participants did not know which field was going to be lit. The stimulus to return to the center square was predictable, since the athletes were instructed that they were always to return to the center field. As these running times may be away from and toward the center plate might be different, we calculated both the average split times for movements towards the outside plates (Split_Out_) and the average split times for movements back to the center plate (Split_In_). Exemplary sequences of the SAT and the CAT are presented in Figure 1. For all tests, we chose the trial with the fastest total time (TT) as the primar performance outcome. For SAT and CAT, Split_In_ and Split_Out_ served as additional performance outcomes.

For purposes of evaluating test-retest reliability and participant habituation, participants performed the whole protocol twice within one week with at least a 48-hour interval between sessions. Before testing, each participant performed a standardized warm-up. In the first phase of the warm-up, participants ran on the SpeedCourt at a moderate intensity for three minutes while changing their direction according to the fields lit on the screen. During the second phase of the warm-up, participants were familiarized with the COD, SAT and CAT. Participants performed all test trials twice.

### Statistical Analyses

To disentangle the effects of cognitive complexity of the outcome measures, we applied several statistical tests. To analyze the effects of stimulus complexity (SAT vs. CAT) and session day (Session 1 vs. Session 2) on agility performance, we performed a 2 (Sessions: I and II) x 2 (Complexity: SAT and CAT) analysis of variance (ANOVA) for which the dependent variables of interest were total time (TT), time out (Split_OUT_) and time in (Split_IN_). To analyze the intra-individual relationships between participant performances on the SAT and the CAT, we calculated Pearson correlation coefficients (r) using performance outcomes of Session II after verification of the normal distribution of the datasets. To assess test-retest reliability between the two test sessions, we calculated Intraclass-Correlation-Coefficients (ICC), based on single ratings, absolute agreement and a 2-way mixed effects model. The ICC serves as a measure of relative reliability whose values range between 0 and 1, where 1 indicates perfect agreement between two measurements (Koo & Li, 2016). ICCs were calculated for COD, SAT and CAT. Next regarding relative reliability, we applied absolute reliability by means of the standard error of measurement (SEM) as a measure of absolute changes between two given measurements. Based on the ratio between the SEM and the mean values of the given outcome, we calculated the coefficient of variation (CoV) in percentage (Hopkins et al., 2001). The alpha level for significance was set at *p*<.05 for all statistical tests. ICC values >0.7 and CoV values <5% were defined as acceptable (Atkinson & Nevill, 1998; Hopkins et al., 2001). Effect sizes were estimated by calculating partial eta^2^ for ANOVA main-effects (Lakens, 2013). All statistical analyses were performed using customized scripts for MATLAB (Mathworks R2020a).

## Results

The ANOVAs revealed significant main effects for Session (session I/session II) on TT (F (_1,21_)=14.15; *p* < .001; ηp2 = .4), SplitIN (F (_1,21_)=4,89; *p* = .04; ηp2 = .19), and SplitOUT (F (_1,21_)=11.86, *p* = .002, ηp2 =.36). We also observed main-effects for Complexity (SAT/CAT) for TT (F (_1,21_)=98.14, *p* < .001, ηp2 = .82), SplitIN (F (_1,21_)=11.52, *p* = .003, ηp2 = .35), and SplitOUT (F (_1,21_)=140.98, *p* < .001, ηp2 =.87). Post-hoc analyses revealed significantly reduced times during Session II as compared to Session I for all analyzed outcome variables. Regarding neurocognitive complexity, there was significantly reduced performance on the CAT compared to the SAT. Moreover, there were significant interaction effects between Session and Complexity for TT (F (_1,21_)=13.04, *p* = .002, ηp2 = .38) and SplitOut. (F (_1,21_)=5.67, *p* = .03, ηp2 = .21). Post-hoc t-tests revealed that TT (*p* < .01) and SplitOut (*p* < .01) decreased significantly from session I to session II for the CAT, but not for the SAT. An overview of the results of this ANOVA, including *p*-values and effect size estimates is provided in Table 1.

**Table 1.** Overview of ANOVA Statistics Resulting from the Comparison of Outcomes on a Simple and a Complex Agility Test, Each Performed Twice Within 1 Week.

		Session	TT	In	Out
			*M* (SD)	*M* (*SD*)	*M* (*SD*)
Results	SAT	I	18.64 (1.1)	1.29 (0.2)	1.72 (0.2)
		II	18.33 (1.0)	1.26 (0.1)	1.68 (0.2)
	CAT	I	19.86 (1.1)	1.33 (.2)	1.95 (.3)
		II	19.00 (1.1)	1.29 (.2)	1.82 (.21)
ANOVA	Session	F	14.15	4.89	11.86
		*P*	<.001	**.04**	**.002**
		_part._ eta^2^	0.40	0.19	0.36
	Complexity	F	98.14	11.52	140.98
		*P*	**<.001**	**.003**	**<.001**
		_part._ eta^2^	0.82	0.35	0.87
	Interaction	F	13.04	.36	5.67
		*P*	**.002**	.50	**.03**
		_part._ eta^2^	0.38	0.02	0.21

*Note*. Session and task complexity are factors in the repeated measures model. Significant effect (*p* < .05) on total time (TT), average split time for inside movement (In) and average split time for outside movements (Out) are marked in bold font.

**Figure 4.**
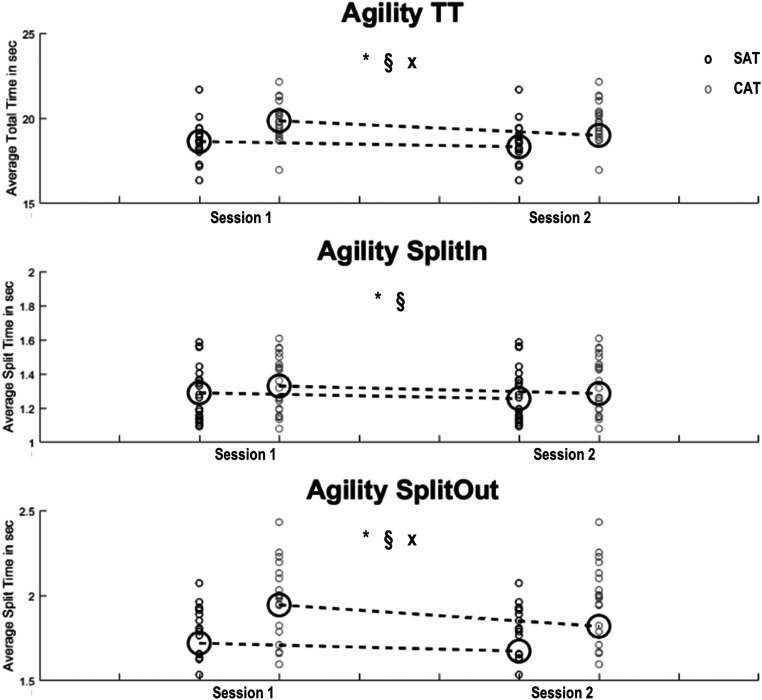
Overview of Differences in Agility Performance When Comparing a Simple (SAT) and Complex (CAT) Agility Test. *Note*. Scatter plots display distribution of performance of 22 participants in the SAT (small black circles) and CAT (small grey circles), including mean values (big black circles). Dashed lines indicate mean changes from session to session. Outcomes analyzed were agility total time (TT), average split time for inside movements (SplitIn) and average split time for outside movement (SplitOut). * = significant main effect for complexity (SAT vs. CAT), § = significant main effect for session (Session 1 vs. Session 2), x = significant complexity x session interaction effect. Level of significance was set at *p* < .05.

Analysis of relative reliability revealed significant relative ICC values for all parameters when comparing Sessions I and II. The lowest ICC values were observed for COD_TT_ (.58), whereas highest values were observed for SAT Split_In_ (.90). In general, SAT revealed higher ICC values as compared to CAT. Regarding absolute reliability, CoV values remained good (<5%) for all outcomes despite CAT Split_Out_ (5.37%). Lowest CoV values were observed for SAT_TT_ (2.45%); the highest value was observed for CAT Split_Out_. Table 2 and Figure 4 provide overviews of these results.

**Table 2.** Overview of ICC Values Revealed from Three Different Tests: Change of Direction Speed, Simple Agility Test and Complex Agility Test.

Test	Variable	Session I	Session II	ICC [LB UB]	SEM [LB UB]	CoV (%) [LB UB]
		*M* (*SD*)	*M* (*SD*)			
COD	TT	8.95 (0.5)	8.54 (0.5)	.60 [.04 -.83]	.31 [.47-.20]	3.53 [5.4–2.28]
SAT	TT	18.64 (1.1)	18.33 (1.0)	.79 [.5 -.91]	.46 [.71-.30]	2.49 [3.83–1.61]
	In	1.29 (0.2)	1.26 (0.1)	.91 [.78 -.96]	.04 [.07 -.03]	3.50 [5.39–2.26]
	Out	1.72 (0.2)	1.68 (0.2)	.89 [.75 -.96]	.07 [.11- .05]	4.08 [6.29–2.64]
CAT	TT	19.86 (1.1)	19.00 (1.1)	.70 [.29 -.88]	.61 [.94 -.40]	3.13 [4.84–2.03]
	In	1.33 (.2)	1.29 (.2)	.87 [.70 -.95]	.06 [.09 -.04]	4.32 [6.65–2.79]
	Out	1.95 (.3)	1.82 (.2)	.80 [.52 -.92]	.10 [.16 -.07]	5.54 [8.53–3.58]

*Note*. For COD, SAT, and CAT, best total time (TT) is provided. For SAT and CAT, average split times for inside movements (In) and average split times for outside movements (Out) are provided. ICCs are presented with lower (LB) and upper bounds (UB) of 95% confidence intervals. The provided *p*-values indicate the level of significance of the correlation analysis.

## Discussion

Our main finding in the present study was that higher order cognitive demands affected the measurement of agility performance on the SpeedCourt when compared to measurements based only on tests with lower order cognitive demands. As would be expected, there were differences in female athletes’ motor performance times on simple (SAT) and complex (CAT) cognitively demanding agility tests. Both the CAT and SAT tests we designed showed acceptable absolute and relative reliability. By adding the more cognitively complex elements of working memory and response inhibition to the simpler reaction time cognitive tasks, we found that measurements of agility performance differed significantly. Interestingly, this was not only the case for total time and for the average outside split times (when athletes were running toward unpredictably changing spots on a grid) but also for average inside split times (when the athletes were predictably running to “home” plate when there were no task differences in cognitive complexity).

These reduced overall and split performance times suggest a motor-interference effect from increased cognitive load. This is in line with conclusions from a previous review on jumping and sidestepping kinematics that highlighted deviations in kinematics when participants performed motor tasks with additional cognitive demands (Brown et al., 2014). Also, Henry et al. (2012) observed reduced agility performance when feints were presented to athletes in a reactive agility paradigm. The interfering effect of cognitive load on motor behavior was also reported in a review of dual-task investigations in athletes (Moreira et al., 2021); these authors found that cognitive demands interfered with motor execution such that performance was impaired on both motor and cognitive sub-tasks by reducing perception-action-coupling capabilities. Moderating variables for these dual-task costs were assigned to the individual’s working memory capacity, and the complexity of the cognitive respectively motor task (Moreira et al., 2021). The higher the demands of coexistent tasks, the higher the likelihood of ‘performance choking,’ defined as a reduction of athletic performance (Moreira et al., 2021). Accordingly, the reduced agility performance we documented in the present study might reflect “choking” induced by increased task-complexity of the coexistent cognitive task. Since no study to date has analyzed agility with direct measures of increasing cognitive demands, we also tested the participants’ simple COD performance to permit a comparison of our participant cohort with participant cohorts in previous studies. Our female participants’ COD speed appeared similar to male participants in Düking et al. (2015) who ran a shorter distance. However, participants’ differential expertise may further modulate the effect of cognitive demands on agility performance (Pojskic et al., 2018; Sekulic et al., 2019; Spasic et al., 2015).

Since cognitive affordances during match play typically go beyond reactive processing to proactive and anticipatory cognitive processes (Huijgen et al., 2015; Vaeyens et al., 2007), COD tasks and simple reactive agility tasks used in most prior research failed to reflect real-world agility behavior. To realistically assess qualitative and quantitative correlates of agility performance, we recommend implementing complex and ecologically valid cognitive elements into agility testing and training. For instance, dynamic stimuli, as described by Lee et al. (2013), in which participants interact with human stimuli, might advance the assessment of agility in complex but controlled paradigms using systems like the SpeedCourt (Lee et al., 2013).

The significant improvements in performance for all agility outcomes that we observed between participants’ first and the second efforts are in line with previous reported findings (Krolo et al., 2020; Sporis et al., 2010). In one study, investigators used a third assessment day and found no further improvements in agility performance beyond this initial habituation to the task (Sporis et al., 2010). In complex tests, habituation effects that result in short-term motor learning may be evident. Therefore, future studies assessing agility performance with cognitive stimuli should consider repeated measurements to control for habituation effects and better determine true meaningful changes in longitudinal performance assessments.

Beyond significantly different performance on sessions one and two, we observed excellent absolute and relative reliability for all CAT and SAT outcome variables, as seen by ICCs and CoV estimates. Predictably, the SAT demonstrated slightly higher reliability when compared to the CAT. According to Krolo et al. (2020), complex tests are more likely to show reduced correlation, since each sub-determinate of performance, in this case motor skills, perceptual-cognitive abilities and technical skills, theoretically generates a separate source of measurement error from day to day (Krolo et al., 2020). Since motor and technical affordances were similar for both SAT and CAT, the increased complexity of the CAT might be treated as a possible reason for reduced reliability. Thus, these observations may indicate that even complex agility tests show satisfactory absolute and relative reliability, even if test-retest reliability decreases with greater cognitive challenges. As noted, our female athletes’ COD speed was similar to what Düking et al. (2016) found with males athletes running a shorter distance.

### Limitations and Directions for Further Research

Despite its novel insights into agility assessment, our study has important limitations when interpreting these findings. The chief concern is that our participant sample was restricted to a small group of female athletes. Sekulic et al. (2013) revealed sex-related-differences not only in performance, but also in sub-determinant contributions to agility performance (Sekulic et al., 2013). Therefore, our findings can only be very cautiously generalized to male participants and other groups. As expertise has also been found to moderate agility performance (Krolo et al., 2020; Pojskic et al., 2018; Sekulic et al., 2019), future studies should use larger and more diverse samples with respect to *both* sex and expertise.

Secondly, the CAT, with its multidirectional motor demands and complex cognitive affordances might be a good example of an agility test with increased ecological validity. But to gain valuable insights into athletes’ agility performance, new tests need to be developed that address other demands of motor and technical skills and of cognitive abilities that are similar to game challenges during team sports competitions. The CAT and other such real-word athletic tasks of EF should be correlated with more traditional laboratory-based neuropsychological tests of EF to help determine whether they, in fact, measure the EF construct. Additionally, in keeping with the perspective in this study, coaches and researchers must be aware that agility tests restricted to lower order cognitive tasks may not adequately simulate these competitive situations, and they can overestimate agility performance. Especially for preparation and rehabilitation purposes, the environmental cues applied in agility setups should induce similar processes of perception-action coupling as those experienced during match play. This would allow practitioners to close the gap from isolated training/therapy towards a sport-specific context in a controlled environment by a stepwise increase of the complexity of agility demands. Keeping in mind that basic cognitive processes may contribute to agility performance, future studies may involve standardized cognitive tests as part of the athlete´s assessment (Scharfen & Memmert, 2019). This would allow future investigators to decompose agility into motor and cognitive components and it would provide complementary data to that of existing studies that restricted analyses of athletic component predictors of agility (Sekulic et al., 2013)

## Conclusion

In the present study, we revealed that agility performance – expressed by agility time - decreased when associated cognitive demands increased. These findings are in line with previous research indicating that cognitive load may interfere with motor performance (Moreira et al., 2021) and kinematics (Brown et al., 2014). Importantly, we also demonstrated good to excellent reliability for the CAT test, suggesting its utility in future studies of this kind. Meanwhile, coaches may use these new insights to tailor more controlled but ecological valid training environments or test-setups for improving agility in team sports.
